# Differential associations of exercise intensity, duration, and frequency with smartphone addiction: the mediating roles of mindfulness and anxiety

**DOI:** 10.3389/fpsyg.2026.1793843

**Published:** 2026-04-13

**Authors:** Huangkun Chen, Yanhao Wang, Jingyou Zhong, Hongliang Wu, Ming Li

**Affiliations:** School of Physical Education and Sport Science, Fujian Normal University, Fuzhou, China

**Keywords:** anxiety, exercise duration, exercise frequency, exercise intensity, mindfulness, physical exercise, smartphone addiction

## Abstract

**Objective:**

This study adopts a specificity perspective of different dimensions of physical exercise to examine the relationships between exercise intensity, duration, and frequency and smartphone addiction among college students, as well as the underlying psychological mechanisms. It further investigates the mediating roles of mindfulness and anxiety, with particular attention to the differences in how these exercise dimensions operate within the model.

**Methods:**

Using stratified random sampling, 1,795 college students in Fuzhou, Fujian Province, China, were surveyed. Physical exercise intensity, duration, and frequency were measured using the Physical Activity Rating Scale-3 (PARS-3), while mindfulness, anxiety, and smartphone addiction were assessed using the Mindful Attention Awareness Scale (MAAS), the Self-Rating Anxiety Scale (SAS), and the Smartphone Addiction Scale–Short Version (SAS-SV), respectively.

**Results:**

(1) All three dimensions of physical exercise—intensity, duration, and frequency—significantly and negatively predicted smartphone addiction (total effects: intensity = −0.139, duration = −0.119, frequency = −0.096; all *p* < 0.01). (2) Significant direct effects were found for intensity and duration (*β* = −0.083 and −0.080, respectively; *p* < 0.01), but not for frequency (*β* = −0.019, *p* > 0.05). (3) Mindfulness mediated the associations across all three dimensions; anxiety mediated only the intensity pathway; and the chain mediation from mindfulness to anxiety was significant for all dimensions.

**Conclusion:**

Different dimensions of physical exercise show distinct associations with smartphone addiction among college students. By examining exercise intensity, duration, and frequency separately rather than treating physical activity as a single activity-level indicator, this study provides a new perspective for understanding the psychological mechanisms linking physical exercise and smartphone addiction.

## 1 Introduction

With the widespread use of smartphones, smartphone addiction has become increasingly prevalent among Chinese university students and is associated with poorer academic performance, impaired social functioning, sleep disturbances, and negative emotional outcomes (Liu et al., 2017; Elhai et al., 2017). Consistent evidence indicates a negative association between physical exercise and smartphone addiction, with psychological variables such as self-control and self-esteem identified as mediators (Kim et al., 2015; Yang et al., 2019; Ke et al., 2024).

Mindfulness and anxiety are key psychological correlates of smartphone addiction. Higher mindfulness is associated with better self-regulation and lower addictive tendencies (Garland et al., 2013; Elhai et al., 2019), whereas anxiety is positively associated with compensatory smartphone use (Billieux et al., 2015; Elhai et al., 2017). Evidence further suggests that improvements in mindfulness may precede reductions in anxiety (Segal et al., 2002; Baer et al., 2006). Physical exercise has been linked to both increased mindfulness (Guo, 2025) and reduced anxiety (Herring et al., 2010).

However, most prior research has treated physical exercise as a single composite construct, rarely distinguishing exercise intensity, duration, and frequency. These dimensions differ in physiological activation and behavioral stability (Caspersen et al., 1985; Schmid et al., 2014), which may imply distinct psychological regulatory processes.

Exercise intensity involves acute physiological arousal and executive engagement (Diamond, 2013; Biddle et al., 2019) and has been associated with short-term anxiety reduction (Herring et al., 2010). Exercise duration reflects sustained participation and continuous attentional involvement (Cox et al., 2018), conceptually aligned with awareness processes described in mindfulness theory (Kabat-Zinn, 2003). Exercise frequency represents behavioral regularity and habit formation (Lally and Gardner, 2011), which has been associated with improved symptom stability and emotional regulation through regular physical activity (Stubbs et al., 2018).

Taken together, these distinctions suggest that exercise intensity, duration, and frequency may relate differently to mindfulness and anxiety, and therefore may demonstrate differentiated psychological pathways in their associations with smartphone addiction. Nevertheless, existing research has not examined these three dimensions simultaneously within a single structural framework.

Accordingly, the present study adopts a dimensional perspective of physical exercise, distinguishing exercise intensity, duration, and frequency within a single analytical framework. By comparing their structural pathways to smartphone addiction and examining the mediating roles of mindfulness and anxiety, this study aims to clarify how different exercise characteristics may operate through differentiated psychological mechanisms.

Based on the theoretical framework, the following hypotheses were proposed:

H1. Exercise intensity, duration, and frequency are negatively associated with smartphone addiction.

H2. Mindfulness mediates the relationships between different exercise dimensions and smartphone addiction.

H3. Anxiety mediates the relationships between different exercise dimensions and smartphone addiction.

H4. Mindfulness and anxiety form a sequential mediating pathway between different exercise dimensions and smartphone addiction.

H5. The effects of exercise intensity, duration, and frequency on smartphone addiction differ in magnitude and underlying mediation patterns.

**Figure 1:**
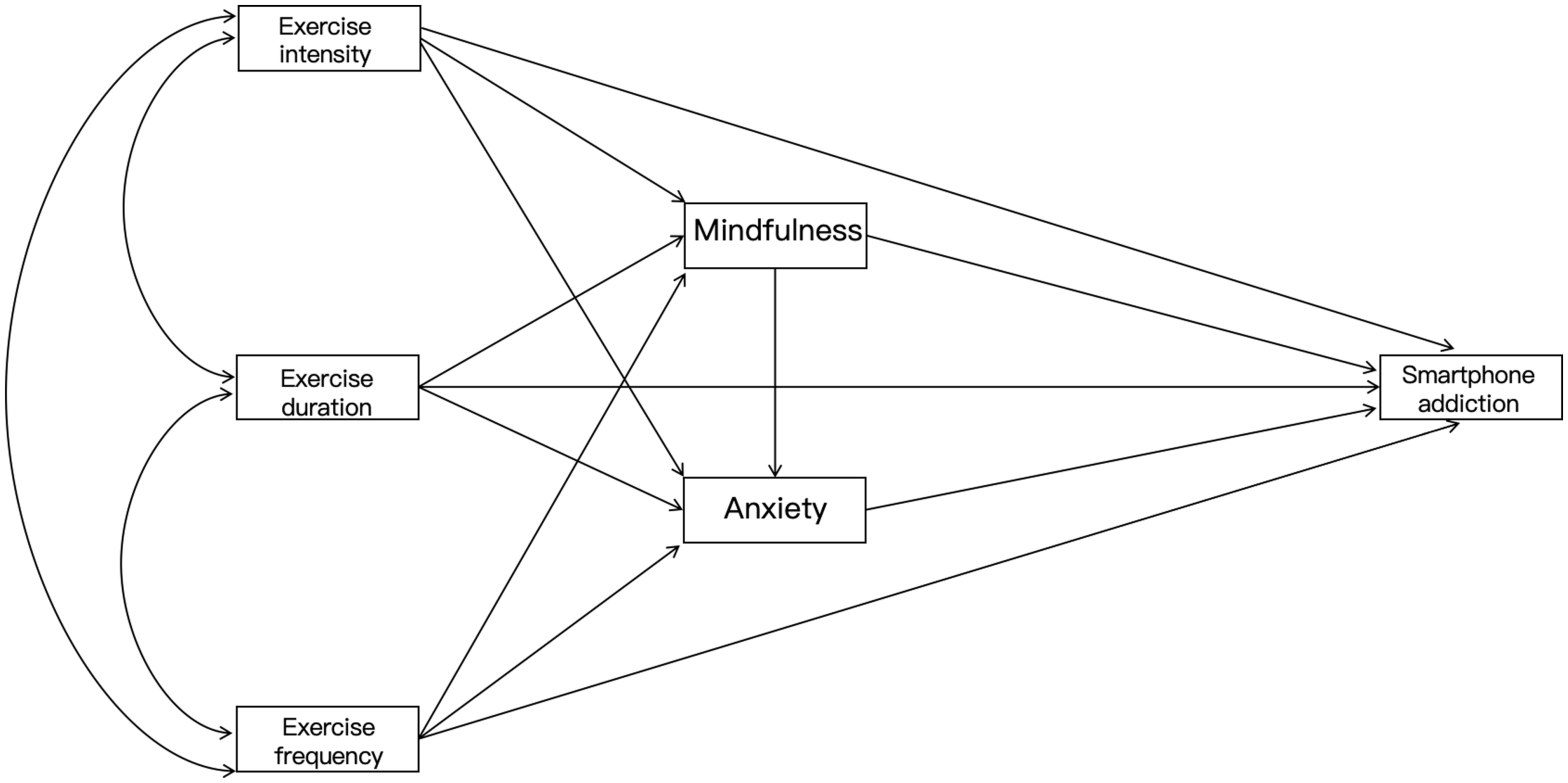
Conceptual model depicting the hypothesized pathways linking exercise intensity, duration, and frequency with smartphone addiction via mindfulness and anxiety.

The proposed hypothesized model is presented in Figure 1.

## 2 Research methods

### 2.1 Participants

*A priori* power analysis was conducted using G*Power 3.1. Assuming a small-to-moderate effect size (*f*^2^ = 0.02), *α* = 0.05, and power = 0.95, the required minimum sample size was 995. The final sample size (*N* = 1,670) exceeded this threshold, indicating sufficient statistical power. Participants were undergraduate students recruited from universities in Fuzhou, Fujian Province, China. A stratified random sampling method was used to conduct a questionnaire survey among 1,795 students. After excluding incomplete responses, 1,670 valid questionnaires were retained, yielding an effective response rate of 93.04%. Participants ranged in age from 18 to 22 years and were undergraduate students from first to fourth year, including 682 male and 988 female students.

### 2.2 Research procedure

This study was conducted in accordance with established ethical guidelines, with full protection of participants’ rights and privacy. Prior to data collection, approval was obtained from the relevant institutional authorities. All participants were informed of the study purpose and procedures and were advised that their participation was entirely voluntary, with the right to withdraw at any time without penalty. Confidentiality of all collected data was strictly ensured, and the questionnaire was administered anonymously to reduce potential social desirability bias.

### 2.3 Physical activity rating scale (PARS-3)

This study used the Physical Activity Rating Scale (PARS-3) (Liang, 1994) to assess exercise intensity, exercise duration, and exercise frequency. As the scale comprises conceptually distinct behavioral dimensions rather than multiple homogeneous items, the three indicators were analyzed separately.

### 2.4 Mindful attention awareness scale (MAAS)

Mindfulness was measured using the Chinese version of the Mindful Attention Awareness Scale (MAAS) revised by Chen et al. (2012). The MAAS is a unidimensional scale comprising 15 items rated on a six-point Likert scale. Higher total scores reflect higher levels of dispositional mindfulness. In this study, the scale demonstrated good internal consistency, with a Cronbach’s *α* coefficient of 0.899.

### 2.5 Self-rating anxiety scale (SAS)

Anxiety was assessed using the Self-Rating Anxiety Scale (SAS) developed by Zung (1971). The Chinese version revised by Wang and Chi (1984) was employed in the present study. The scale consists of 20 items rated on a four-point Likert scale, with Items 5, 9, 13, 17, and 19 reverse-coded. Item scores are summed to obtain a raw score, which is then multiplied by 1.25 to yield a standard score. Higher scores indicate higher levels of anxiety, with a standard score of 50 or above suggesting the presence of clinically relevant anxiety symptoms. In the present sample, the Cronbach’s *α* coefficient was 0.814.

### 2.6 Smartphone addiction scale–short version (SAS-SV)

Smartphone addiction was assessed using the Smartphone Addiction Scale–Short Version (SAS-SV) developed by Kwon et al. (2013). The Chinese version translated and validated by Zhao et al. (2022) was used. The scale contains 10 items rated on a six-point Likert scale, with higher total scores indicating greater severity of smartphone addiction tendencies. In the present study, the Cronbach’s *α* coefficient was 0.901.

### 2.7 Statistical analyses

Data were analyzed using SPSS 27.0. Preliminary analyses included tests for common method bias, descriptive statistics, normality assessment, correlation analysis, and multicollinearity diagnostics. Given the relatively large number of variables and the complexity of the hypothesized paths, a path analysis approach within the structural equation modeling (SEM) framework was adopted using observed variables only (Wen and Ye, 2014). Specifically, AMOS 26.0 was used to construct a path analysis model with observed variables, and parameters were estimated using the maximum likelihood (ML) method. Mediation effects were tested using bias-corrected nonparametric percentile bootstrap procedures with 5,000 resamples, and 95% confidence intervals were calculated to evaluate the significance of indirect effects.

## 3 Results and analysis

### 3.1 Common method bias test

To assess potential common method bias, Harman’s single-factor test was conducted using exploratory factor analysis. The results extracted six factors with eigenvalues greater than 1. The first factor accounted for 29.913% of the total variance, which was below the commonly used threshold of 40% (Podsakoff et al., 2003). These findings suggest that common method bias was unlikely to be a serious concern in the present study.

### 3.2 Descriptive statistics and correlation analysis

The descriptive statistics and Pearson correlation analysis of the main variables are presented in Table 1. The results showed that the dimensions of physical exercise were significantly positively correlated with each other (*p* < 0.01). Further analyses indicated that the dimensions of physical exercise were significantly positively correlated with mindfulness (*p* < 0.01) and significantly negatively correlated with anxiety (*p* < 0.01) and smartphone addiction (*p* < 0.01). Mindfulness was significantly negatively correlated with anxiety (*p* < 0.01) and smartphone addiction (*p* < 0.01), whereas anxiety was significantly positively correlated with smartphone addiction (*p* < 0.01). The significant correlations among the main variables provide a basis for subsequent mediation analyses.

**Table 1:** Results of descriptive statistical analysis and correlation analysis of variables (*N* = 1,670).

Variable	M ± SD	Exercise intensity	Exercise duration	Exercise frequency	Mindfulness	Anxiety	Smartphone addiction
Exercise intensity	2.5 ± 1.1	1					
Exercise duration	2.77 ± 1.16	0.521**	1				
Exercise frequency	3.13 ± 0.99	0.302**	0.241**	1			
Mindfulness	60.3 ± 13.23	0.212**	0.164**	0.221**	1		
Anxiety	45.88 ± 9.11	−0.197**	−0.155**	−0.146**	−0.420**	1	
Smartphone addiction	29.76 ± 9.97	−0.232**	−0.208**	−0.152**	−0.370**	0.408**	1

*At 0.05 level (two tails), the correlation is significant. **At 0.01 level (two-tailed), correlation is significant.

### 3.3 Multicollinearity diagnostics

Given the moderate correlation observed between exercise intensity and exercise duration (*r* = 0.52), a potential risk of multicollinearity was considered. To evaluate whether this association could bias parameter estimation, multicollinearity diagnostics were conducted (Table 2). The results indicated that the tolerance values and variance inflation factors (VIFs) for all predictors were within acceptable ranges, suggesting no serious multicollinearity concerns.

**Table 2:** Multiple regression diagnostics for smartphone addiction.

Variable	Unstandardized coefficient (*B*)	Standard error	Standardized coefficient (*β*)	*t*	*p*	Tolerance	VIF
Constant	29.484	1.986	–	14.846	<0.001	–	–
Exercise intensity	−0.730	0.228	−0.083	−3.196	0.001	0.682	1.466
Exercise duration	−0.663	0.210	−0.080	−3.161	0.002	0.718	1.392
Exercise frequency	−0.182	0.223	−0.019	−0.816	0.415	0.875	1.143
Mindfulness	−0.157	0.018	−0.214	−8.883	<0.001	0.789	1.268
Anxiety	0.304	0.025	0.287	12.029	<0.001	0.81	1.235

The dependent variable was smartphone addiction. VIF = variance inflation factor; tolerance.

To further assess the adequacy of the regression assumptions, residual diagnostics were performed (Table 3). The mean of the residuals was close to zero, with a standard deviation of 8.429. The standardized residuals also showed a mean of 0 and a standard deviation close to 1 (0.998), consistent with the assumption of normality. The range of standardized residuals (−3.618 to 3.759) did not indicate the presence of extreme outliers (|*z*| > 3), suggesting a reasonable residual distribution.

**Table 3:** Residual statistics of the regression model (*N* = 1,670).

Statistic	Minimum	Maximum	Mean	Standard deviation
Predicted value	15.26	45.45	29.76	4.752
Residual	−30.547	31.737	0	8.429
Standardized predicted value	−3.053	3.303	0	1
Standardized residual	−3.618	3.759	0	0.998

The dependent variable was smartphone addiction.

### 3.4 Mediation effect test

**Figure 2:**
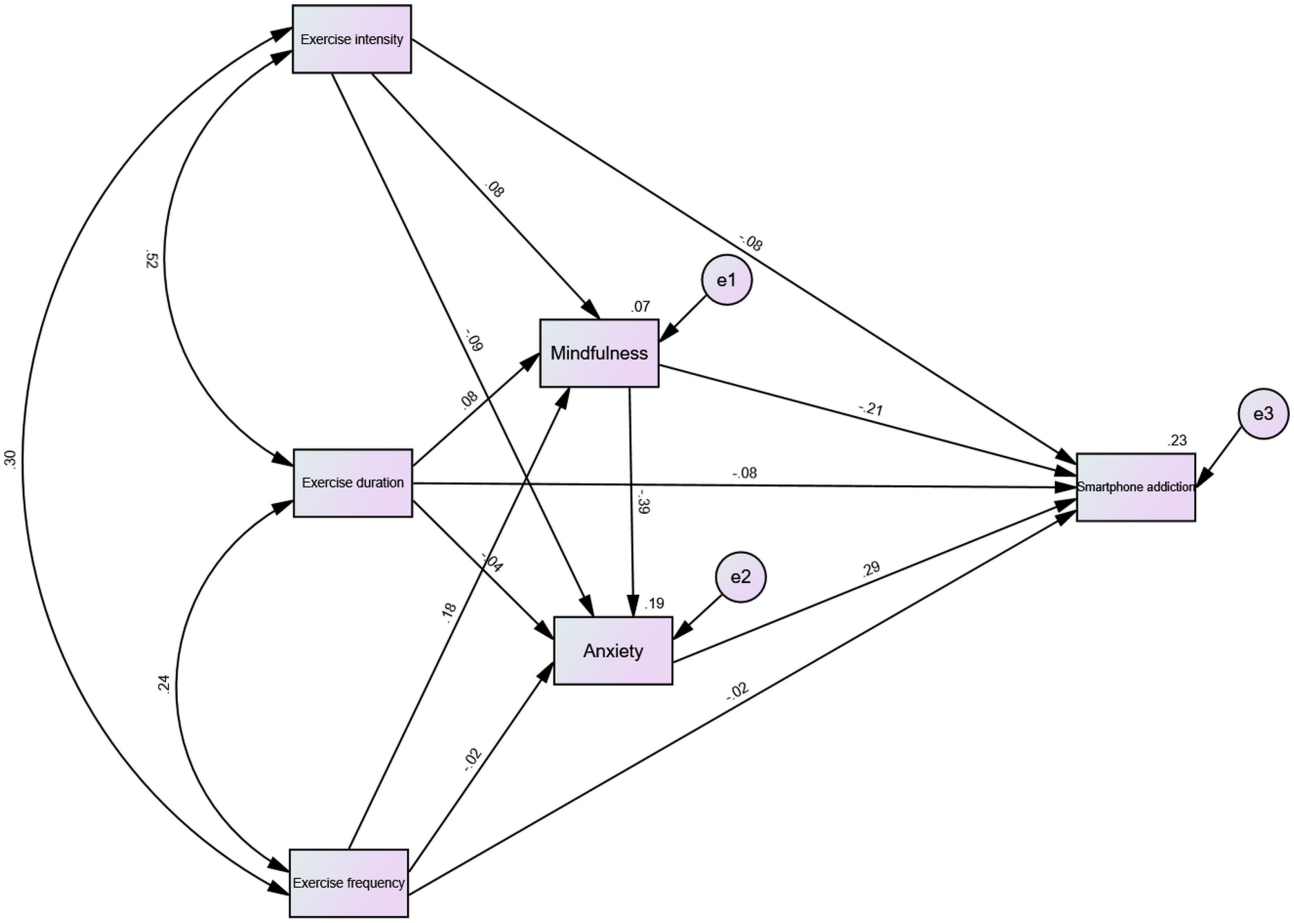
Path analysis diagram of the theoretical hypothesized model.

To examine whether different dimensions of physical exercise were associated with smartphone addiction through mindfulness and anxiety, a structural equation model was constructed using AMOS 26.0 (see Figure 2). Mediation effects were tested with a bias-corrected percentile bootstrap procedure based on 5,000 resamples and 95% confidence intervals.

The proposed model showed a good fit to the data: CMIN/DF = 1.435, CFI = 0.999, TLI = 0.980, NFI = 0.998, IFI = 0.999, RMSEA = 0.036, and SRMR = 0.0085, indicating that the hypothesized structural model was well supported by the observed data.

#### 3.4.1 Exercise intensity

As shown in Table 4, exercise intensity was significantly and negatively associated with smartphone addiction (*β* = −0.083, 95% CI [−0.135, −0.029], *p* < 0.05), accounting for 50.3% of the total effect. In addition, the total indirect effect through mindfulness and anxiety was also significant (*β* = −0.082, 95% CI [−0.070, −0.034], *p* < 0.001).

**Table 4:** Mediation analysis of exercise intensity on smartphone addiction.

Pathway	Standardized estimate (*β*)	95% CI	*p*	Effect proportion (%)
Direct effect	−0.083	[−0.135, −0.029]	0.002	50.3
Total indirect effect	−0.082	[−0.070, −0.034]	<0.001	49.7
Exercise intensity → Mindfulness → Smartphone addiction	−0.038	[−0.052, −0.025]	<0.001	23.0
Exercise intensity → Anxiety → Smartphone addiction	−0.025	[−0.041, −0.010]	0.003	15.2
Exercise intensity → Mindfulness → Anxiety → Smartphone addiction	−0.019	[−0.028, −0.012]	<0.001	11.5
Total effect	−0.165	[−0.187, −0.080]	<0.001	100

CI = confidence interval.

Path-specific analyses indicated that mindfulness functioned as a significant mediator between exercise intensity and smartphone addiction. Moreover, the serial mediation pathway involving mindfulness and anxiety was statistically significant, accounting for 11.5% of the total effect. These findings indicate that the observed statistical associations between exercise intensity and smartphone addiction may involve both direct and indirect pathways through mindfulness and anxiety.

The total statistical association of exercise intensity with smartphone addiction was significant (*β* = −0.165, *p* < 0.001).

#### 3.4.2 Exercise duration

As presented in Table 5, exercise duration showed a significant direct association with smartphone addiction (*β* = −0.080, 95% CI [−0.128, −0.031], *p* < 0.05), accounting for 67.2% of the total effect.

**Table 5:** Mediation analysis of exercise duration on smartphone addiction.

Pathway	Standardized effect (*β*)	95% CI	*p*	Effect proportion (%)
Direct effect	−0.080	[−0.128, −0.031]	0.002	67.2
Total indirect effect	−0.039	[−0.059, −0.016]	0.001	32.8
Exercise duration → Mindfulness → Smartphone addiction	−0.017	[−0.029, −0.006]	0.002	14.3
Exercise duration → Anxiety → Smartphone addiction	−0.012	[−0.029, 0.004]	0.13	10.1
Exercise duration → Mindfulness → Anxiety → Smartphone addiction	−0.010	[−0.017, −0.003]	0.002	8.4
Total effect	−0.119	[−0.170, −0.065]	<0.001	100

CI = confidence interval.

The total indirect effect of exercise duration was also significant, although smaller in magnitude (*β* = −0.039, *p* < 0.05). Examination of individual pathways revealed that this indirect association was primarily transmitted through mindfulness. In contrast, the mediation pathway via anxiety alone did not reach statistical significance.

Although the serial mindfulness–anxiety pathway was statistically significant, its contribution to the total effect was relatively modest. The overall effect of exercise duration on smartphone addiction was −0.119 (*p* < 0.001).

#### 3.4.3 Exercise frequency

As shown in Table 6, exercise frequency was not directly associated with smartphone addiction (*β* = −0.019, *p* = 0.458).

**Table 6:** Mediation analysis of exercise frequency on smartphone addiction.

Pathway	Standardized effect (*β*)	95% CI	*p*	Effect proportion (%)
Direct effect	−0.019	[−0.068, 0.031]	0.458	19.8
Total indirect effect	−0.077	[−0.089, −0.043]	<0.001	80.2
Exercise frequency → Mindfulness → Smartphone addiction	−0.038	[−0.052, −0.025]	<0.001	39.6
Exercise frequency → Anxiety → Smartphone addiction	−0.007	[−0.020, 0.007]	0.329	7.3
Exercise frequency → Mindfulness → Anxiety → Smartphone addiction	−0.032	[−0.043, −0.020]	<0.001	33.3
Total effect	−0.096	[−0.134, −0.031]	0.002	100

CI = confidence interval.

In contrast, indirect pathways played a predominant role. The total indirect effect was significant (*β* = −0.077, 95% CI [−0.089, −0.043], *p* < 0.001), accounting for approximately 80% of the total effect. Specifically, exercise frequency was negative related to smartphone addiction mainly through mindfulness and the serial mindfulness–anxiety pathway, whereas the anxiety-only mediation pathway was not significant.

The total effect of exercise frequency on smartphone addiction was −0.096 (*p* < 0.05), indicating that its association with smartphone addiction was largely indirect in nature.

#### 3.4.4 Summary of hypothesis testing

At the level of total effects, exercise intensity, duration, and frequency were all negatively associated with smartphone addiction, supporting Hypothesis 1. However, dimension-specific differences emerged in the structural pathways: significant direct effects were observed for exercise intensity and duration, whereas exercise frequency showed no significant direct association.

Mindfulness significantly mediated the relationships between exercise dimensions and smartphone addiction, supporting Hypothesis 2. All three exercise dimensions were positively associated with mindfulness, which in turn was negatively associated with smartphone addiction.

Anxiety partially mediated the relationships between exercise dimensions and smartphone addiction, providing partial support for Hypothesis 3. Specifically, the mediating role of anxiety was evident in the exercise intensity pathway but was not consistently observed across all dimensions.

The sequential mediation pathway involving mindfulness and anxiety was significant for all three exercise dimensions, supporting Hypothesis 4. Exercise was associated with higher mindfulness, which was related to lower anxiety and subsequently to lower smartphone addiction.

Taken together, the results indicate systematic differences across exercise intensity, duration, and frequency in terms of direct effects and mediation patterns, thereby supporting Hypothesis 5.

## 4 Discussion

From a dimensional perspective, this study examined the associations of exercise intensity, duration, and frequency with smartphone addiction. Consistent with previous research reporting negative associations between physical activity and smartphone addiction (Kim et al., 2015; Fennell et al., 2017; Yang et al., 2019; Yang et al., 2021), all three exercise dimensions showed significant negative total associations. Building on this overall pattern, the following sections examine how these dimensions differed in their structural pathways and psychological involvement.

### 4.1 Structural differences across exercise dimensions

Previous studies have typically examined physical activity as a global construct, with limited differentiation among its internal dimensions. From a dimensional perspective, different aspects of exercise may operate through distinct pathways. The overall association between physical activity and smartphone addiction may therefore reflect a combination of multiple direct and indirect mechanisms.

Exercise intensity, duration, and frequency demonstrated different patterns within the structural model. Exercise intensity and duration showed significant direct associations with smartphone addiction, whereas the direct path from exercise frequency was not statistically significant. These findings indicate structural differences in the pathways through which different exercise dimensions operate.

In the pathways related to exercise intensity, mindfulness, anxiety, and their sequential effect were all significant. In the pathways related to exercise duration, the mediating effect was primarily driven by mindfulness, while anxiety did not show an independent effect. In the pathways related to exercise frequency, mindfulness and the sequential pathway involving anxiety constituted the primary mechanism. Mindfulness was negatively associated with smartphone addiction, whereas anxiety showed a positive association, consistent with previous research identifying mindfulness as a protective factor and anxiety as a risk factor (Yang et al., 2019; Elhai et al., 2017). These findings suggest that psychological regulatory factors function differently across exercise dimensions.

From an overall structural perspective, exercise intensity and duration were more likely to exert direct effects, whereas exercise frequency primarily operated through psychological variables. According to habit formation theory, behaviors that are repeatedly performed over time tend to develop into habits characterized by a high degree of automaticity (Lally and Gardner, 2011). Compared with exercise intensity or duration, exercise frequency reflects behavioral repetition rather than the psychological engagement involved in a single exercise session. Consequently, its influence on smartphone addiction may occur indirectly through psychological regulatory factors such as mindfulness and anxiety.

### 4.2 Differences in effect composition across exercise dimensions

Beyond differences in pathway structures, clear differences were also observed in the composition of effects across exercise dimensions. Overall, the direct effects of exercise intensity and exercise duration accounted for a substantial proportion of their total associations with smartphone addiction, whereas the influence of exercise frequency was primarily exerted through indirect pathways.

Specifically, for exercise intensity, the direct effect accounted for 59.7% of the total effect. In addition, indirect effects were observed through mindfulness (23.0%), anxiety (15.2%), and the sequential mediation pathway of mindfulness–anxiety (11.5%). These findings suggest that exercise intensity not only exerts a relatively strong direct influence but also affects smartphone addiction indirectly through multiple psychological regulatory processes.

For exercise duration, the proportion of the direct effect was even higher (67.2%). The indirect effects mainly operated through mindfulness (14.3%) and the sequential mindfulness–anxiety pathway (8.4%). Compared with exercise intensity, the indirect effects of exercise duration were more concentrated in awareness-related psychological processes. This suggests that sustained engagement in exercise may influence behavior primarily through enhancing individuals’ attentional awareness and psychological regulation. Previous research has shown that physical activity is positively associated with dispositional mindfulness and self-regulation, which can help individuals become more aware of their behavioral impulses and thereby reduce maladaptive technology use (Roberts and Danoff-Burg, 2010; Cox et al., 2016).

No significant direct association was observed between exercise frequency and smartphone addiction, and its influence was mainly exerted through indirect pathways. Among these, the indirect effect through mindfulness accounted for 39.6% of the total effect, while the sequential mindfulness–anxiety pathway accounted for 33.3%. This pattern indicates that exercise frequency may primarily influence smartphone addiction by gradually shaping psychological regulatory processes.

These differences in effect composition may reflect the distinct behavioral characteristics represented by different exercise dimensions. Exercise intensity and exercise duration are more closely related to the level of physical engagement and time investment in a single exercise session and may therefore produce more immediate influences on psychological states and behavioral regulation. A large body of research has demonstrated that physical exercise can effectively reduce anxiety and improve emotional regulation, thereby decreasing the likelihood of maladaptive behavioral patterns (Petruzzello et al., 1991; Stubbs et al., 2018). In contrast, exercise frequency reflects the repetition of behavior in daily life, and its influence on smartphone addiction may therefore occur indirectly through gradual changes in psychological regulatory processes. Consistent with this perspective, previous studies have found that the relationship between physical exercise and problematic smartphone use is often mediated by psychological factors, such as psychological resilience and perceived stress, rather than by simple behavioral substitution (Zhao et al., 2022).

### 4.3 Theoretical implications

The present study contributes to theory by advancing understanding of how physical exercise operates within psychological regulation systems.

From a dimensional perspective, the findings indicate that physical exercise should not be considered a homogeneous protective factor. Instead, its internal structure carries distinct theoretical significance. The differentiated patterns of direct and indirect effects indicate that treating exercise as a single behavioral index may obscure meaningful variation in its psychological functions.

The findings also highlight the differentiated roles of mindfulness and anxiety within exercise-related regulation processes. While mindfulness consistently functioned as a central mediating mechanism, anxiety participated in a conditional and hierarchical manner. This result extends existing addiction models, particularly the Interaction of Person–Affect–Cognition–Execution (I-PACE) model (Brand et al., 2019), by showing that psychological variables may assume different functional roles depending on behavioral context.

In addition, the study provides structural evidence for a multi-level regulatory framework linking exercise and addictive behavior. The differentiation of exercise dimensions across temporal scales, regulatory levels, and psychological pathways supports a more nuanced conceptualization of behavioral self-regulation and offers a theoretical foundation for structurally informed intervention models.

### 4.4 Practical implications

At the practical level, the findings offer more targeted insights for smartphone addiction intervention.

Rather than focusing solely on overall exercise participation or total activity volume, the results highlight the importance of considering exercise structure. Different exercise dimensions appear to exert their regulatory effects through distinct psychological pathways, suggesting that intervention strategies should not rely on a single indicator of exercise engagement.

Specifically, Exercise intensity may be more closely correlated with immediate behavioral regulation and impulse control, whereas exercise duration and frequency may contribute more strongly to awareness development and emotional regulation. Accordingly, intervention programs may benefit from tailoring exercise design to specific intervention goals.

For example, when the primary aim is to reduce impulsive smartphone use, greater emphasis may be placed on appropriately structured exercise intensity. In contrast, when interventions focus on emotional regulation or awareness cultivation, extending exercise duration or enhancing participation stability may be more effective in promoting sustained psychological adjustment.

Overall, the present findings support conceptualizing physical exercise as a regulatory behavior system with differentiated internal functions. Designing interventions from a dimensional perspective may enhance both the precision and explanatory power of exercise-based strategies for smartphone addiction prevention.

### 4.5 Limitations

First, the present model focused on core psychological mechanisms rather than exhaustively modeling all potential determinants of smartphone addiction, which may influence the interpretation of the findings. For example, conditions that might hinder participation in physical exercise and specific smartphone usage patterns were not assessed. These factors may influence smartphone addiction and should be considered in future research to develop a more comprehensive model.

Second, the sample consisted only of university students from Fuzhou, which may limit the generalizability of the findings due to the restricted sampling scope.

Finally, the data in this study were collected through self-reported measures using a cross-sectional design, which precludes causal inference. Future studies should adopt longitudinal or experimental designs to further examine the relationships among variables.

## 5 Conclusion

This study examined the associations of exercise intensity, duration, and frequency with smartphone addiction among college students, as well as the mediating roles of mindfulness and anxiety. All three exercise dimensions were negatively associated with smartphone addiction, but their pathway structures differed. Exercise intensity and duration showed both direct and indirect associations, whereas exercise frequency was primarily associated indirectly through psychological variables. Mindfulness was a consistent mediator across models, while anxiety showed involvement in specific indirect pathways. These findings clarify the differentiated associations between exercise dimensions and smartphone addiction.

By adopting a dimensional perspective of physical exercise, this study contributes to the literature in several ways. First, it moves beyond treating physical activity as a single composite indicator and instead examines the internal structure of exercise behavior. Second, it compares the structural pathways linking different exercise dimensions to smartphone addiction within a unified framework. Third, it identifies the differentiated roles of mindfulness and anxiety in these pathways, offering clearer insight into the psychological processes linking physical exercise and behavioral addiction.

## Data Availability

The original contributions presented in the study are included in the article/supplementary material, further inquiries can be directed to the corresponding author.
